# Personalised and precision mental health in eating disorders: why routine outcome measurement is key

**DOI:** 10.1186/s40337-025-01290-2

**Published:** 2025-07-10

**Authors:** Amelia Austin, Karina L. Allen

**Affiliations:** 1 Mathison Centre for Mental Health Research & Education, University of Calgary, Calgary, Canada; 2 Department of Community Health Sciences, Cumming School of Medicine, University of Calgary, Calgary, Canada; 3 Centre for Eating and Weight Disorders, Institute of Psychiatry, Psychology and Neuroscience, King’s College London, London, SE5 8AF UK; 4 Eating Disorders Outpatients Service, Maudsley Hospital, South London and Maudsley NHS Foundation Trust, London, SE5 8AF UK

**Keywords:** Anorexia nervosa, Bulimia nervosa, ARFID, Routine outcome measurement, Patient reported outcome, Precision psychiatry, Personalized treatment, Measurement-based care, Electronic health records

## Abstract

For over a decade, the mental health field has been interested in precision treatment using psychopharmacological interventions. More recently, this interest has expanded to include psychotherapy, which is the primary treatment modality for eating disorders. Personalised medicine and precision treatment are also seen as priorities for the eating disorder field by those with lived experience and carers, clinicians and researchers. However, precision treatment necessitates the collection of large amounts of clinical data. Three frameworks exist or have been proposed for the purpose of gathering large-scale routine clinical outcomes in eating disorder services: The International Consortium for Health Outcomes Measurement (ICHOM) eating disorder set, the Australia national minimum dataset, and the Eating Disorders Clinical Research Network. Despite the emergence of these frameworks, challenges exist with implementation. This paper outlines the rationale for the collection of routine outcome data in eating disorder treatment settings, the three existing frameworks proposed, and considerations for implementation and scaling. These include clinical and practice applications, technical aspects, statistics, and contextual factors. We invite attention to our recommendations and collaborative approaches to facilitate progress towards precision treatment in eating disorders.

## Abstract (plain language summary)

Precision treatment, also known as precision medicine, involves tailoring treatment to the individual characteristics of each patient. Precision treatment for eating disorders is seen as a priority by individuals with lived experience, their carers, clinicians and researchers. However, precision treatment depends on large amounts of clinical data being collected. Currently, eating disorder services do not collect the same information from or about patients. There is no large clinical database to inform precision medicine decisions. Three main frameworks have been proposed to support largescale and consistent data collection in eating disorder services: The International Consortium for Health Outcomes Measurement (ICHOM) eating disorder set, the Australian national minimum dataset, and the UK Eating Disorders Clinical Research Network. These frameworks hold promise but there are challenges with applying them. This paper summarises why collecting routine outcome data is important, the three main frameworks proposed, and the factors which may help to progress data collection and precision treatment for eating disorders. We consider clinical, practical, technical, statistical and contextual factors. It is important that progress in this area is collaborative and involves individuals with lived experience, carers, clinicians and researchers.

## Introduction

*Personalised treatment* is a broad term describing the selection, adaptation, and/or adjustment of an intervention to meet the specific needs of an individual, with the goal of improving patient outcomes [18]. Within the field of eating disorders (EDs), personalised treatment has been described as using an individualized approach that tailors care to the unique psychological, physical, and social characteristics of the patient [29]. Priority-setting studies in the ED field show a strong desire among people with lived, clinical, and research experience for more individualized approaches in ED treatment [27]. Personalised treatment in EDs encompasses a range of strategies, such as using a person-centred approach that emphasizes the patient's values and preferences, incorporating progress feedback to guide ongoing adjustments to care, or integrating therapeutic modalities to address co-occurring mental health conditions like anxiety or depression. It may also involve adapting interventions based on the patient's readiness for change [46].

Under the umbrella of personalised treatment, *precision treatment* specifically refers to a data-driven approach that allows for treatment decisions to be made in an algorithmic, empirically derived way [36]. This approach utilizes a variety of patient-specific data to predict which interventions are most likely to be effective for an individual. By analyzing patterns within large, pre-existing datasets of similar patients, precision treatment may help to identify the most appropriate form of tailored, evidence-based treatment for an individual, reducing trial-and-error and enhancing outcomes [8]. In the past, precision mental health primarily focused on psychopharmacological interventions, often under the label precision psychiatry, where the goal was to match medications to individuals based on factors like genetic profiles or neurobiological data. More recently, however, there has been a broader recognition that precision approaches should be applied beyond medication, including in psychotherapeutic contexts across a wide range of mental health diagnoses [18] and EDs specifically [51]. This shift is especially pertinent to EDs, where recommended first-line treatment approaches are psychotherapeutic in nature [14, 16, 42]).

A key barrier to precision treatment in EDs is the lack of routine, consistent data collection within and across services. Precision mental health requires data-driven decision-making, but ED services vary significantly in which measures they request from patients and at which time points. Many services struggle to collect data at all (e.g., see Mountford et al. [37]; Richards et al. [44]). When data are collected, they may not be systematically analyzed. These challenges are understandable in an over-stretched healthcare system, where limited resources and high patient volumes may make comprehensive data collection and analysis difficult, particularly following the rise in ED referrals after the COVID-19 pandemic [19]. These issues are not unique to EDs, as they are common across mental health services more broadly (e.g., [50]), but they present a particularly pressing challenge in ED treatment because only 50% of patients (on average) benefit from current ED therapies [47]. Without consistent data, it becomes nearly impossible to monitor treatment progress accurately, predict outcomes with confidence, or tailor interventions to the unique needs of each patient.

Three recent initiatives have emerged to establish more consistent and standardised data collection frameworks within and across ED services. These initiatives, while distinct in their specific goals and priorities, have the potential to shape routine outcome collection in EDs and advance precision mental health approaches.

## Current data collection frameworks for EDs

### International consortium for outcomes measurement (ICHOM)

In 2023, ICHOM published a consensus-based piece on the ideal approach for the routine outcome measurement of EDs [3]. The intention behind the project was to move the field of EDs toward value-based healthcare, with value defined as the ratio between the outcomes achieved and the resources/costs [25]. While building consensus, the ICHOM ED project prioritized the use of patient reported outcomes applicable to all ED diagnoses (although some specificity was permitted for avoidant/restrictive food intake disorder [ARFID]), the harmonization of measurement tools with those used in non-ED mental health settings (i.e., measures used in previous consensus statements for anxiety, depression, obsessive compulsive disorder, and post-traumatic stress disorder [32]), and measurement tools available in multiple languages, at no cost, and with a wealth of published psychometric evidence. The project used a Delphi approach (i.e., iterative rounds of discussion and voting), and included international representatives with professional and/or lived experience. The final report recommended the measurement of multiple baseline/annual characteristics, including demographics, comorbidities (using a checklist approach), and treatment details, and the collection of outcome data related to ED behaviours and cognitions, physical health, co-occurring mental health conditions (including suicidality), and quality of life/social functioning via 3–5 questionnaires determined by age and presenting diagnosis [3].

### Australian national minimum dataset

2023 also saw the publication of a proposed national minimum dataset for Australian ED services [9]. The aim of this project was to create a minimum dataset to better understand the impact of EDs in Australia and support research and quality improvement efforts. This project focused on key domains for assessment, rather than specific outcome measures, but also emphasized the need for consistent outcome assessment. This project used a Delphi approach and included Australian representatives with professional and/or lived experience. The final agreed minimum dataset recommended measurement of demographics, clinical ED diagnosis, comorbidities, medical indicators (e.g., bone health, BMI, weight change, malnutrition diagnosis), treatment characteristics, treatments delivered, and quality of life. Additional ‘tier 2’ data was recommended as optional for collection. With regards to comorbidities, ≥ 90% of all respondents rated substance use disorders, anxiety disorders, suicidality, personality disorders, autism, post-traumatic stress disorder and affective/mood disorders as ‘very important’ or ‘imperative’ to assess in ED patients, followed by non-suicidal self-harm (85%), ADHD (79%) and sleep disturbances (43%). This study also focused on the Australian healthcare settings from where data may be collected, with respondents generally agreeing on the importance of capturing information from ED patients in primary care, private practice (e.g., private outpatient psychological therapy) and specialist ED centres (outpatient and inpatient) [9].

### Eating disorders clinical research network (EDCRN)

The U.K.’s EDCRN is one example of an in-progress project to establish consistent, standardized data collection across ED services [2]. Based at King’s College London and the South London and Maudsley NHS Foundation Trust, EDCRN is jointly steered by people with lived experience and carers, clinicians, and researchers. Consensus building workshops were used to build on the recommendations of ICHOM [3] and the proposed Australian national minimum dataset [9] to agree the variables and measures for collection across child/adolescent and adult U.K. ED services [28]. Core to the EDCRN is development of a digital platform that can collect data in a Trusted Research Environment and provide patients, clinicians, and services with summaries of their information. Additionally, the national EDCRN database will allow for larger scale learning about who is being seen in ED services (and who is not), how current evidence-based treatments are being used, and how these treatments benefit different sub-groups of patients. There will be potential for trial emulation and there is a key emphasis on collection of biological as well as psychosocial variables. This planned work offers hope for significant change in U.K. ED services, but as the project started data collection in early 2025, there is not yet learning available from the work.

## Implementation challenges

The above frameworks represent the first steps towards consistent, widespread routine outcome measurement in ED services. However, a significant challenge will be the successful implementation of these frameworks across healthcare systems and contexts to facilitate a precision approach. Deisenhofer et al. [18] have introduced the Implementing Precision Methods (IPM) Framework, which focuses on the components of the implementation process in psychological treatments. They conceptualise this implementation as consisting of four main processes: clinical and practice applications, technical aspects, statistics, and contextual factors. We use this framework to consider the anticipated barriers and facilitators to routine outcome measurement for precision treatment in EDs, below. We draw on prior research on the implementation of routine outcome monitoring in general mental health settings and also highlight examples of positive practice.

### Clinical and practice applications

Successful implementation will require ‘buy in’ from all stakeholders, but most importantly patients/families and clinicians. Previous studies have found that patients support routine outcome measurement when the purpose is clear and feedback is incorporated into therapy [4], and representatives of people with lived experience of EDs have welcomed the new U.K. EDCRN [20, 28]. However, a precision approach still requires repeated data collection across treatment, up to every session if early change (or lack thereof) is determined to be important to capture. This repeated measurement may feel burdensome to patients and/or families participating in treatment. However, some research demonstrates that the burden diminishes as patients begin to perceive the data collection process as a helpful part of routine clinical care [18]. Further, many people with lived experience of an ED are motivated to engage in research for altruistic reasons (e.g., Dalton et al. [17]), highlighting the potential benefits to future patients and knowledge generation in addition to any benefits they themselves may experience. There has also been a recent move toward passively tracking ED symptoms via wearable technology (e.g., Ralph-Nearman et al. [43]) including during active treatment [33], which may also reduce patient burden in data collection. Such technology requires financial resource, however, either from patients or clinical services and may therefore disadvantage certain individuals and healthcare contexts. Finally, the *process* of data collection needs considering when thinking about the patient experience. Easy-to-use systems (digital or wearable), embedding data collection in routine care, and data being used to inform treatment are all important principles here [5]. Clinicians also have an important role to play in facilitating collaborative and motivational discussions about data, and in making routine outcome measurement part of the therapeutic process rather than something that sits outside of or alongside treatment.

In reference to clinician buy-in, it is important to consider the provision of dedicated resources and time for establishing routine outcome measurement. The Australian national minimum dataset [9] recommends a dedicated individual responsible for data collection in each setting if possible. Aside from time-related concerns, prior research in general mental health settings has found that clinicians may be reluctant to adopt data-informed approaches due to a lack of understanding of potential clinical benefits, concerns about being ‘judged’ or compared to others, and concerns about the ethics of data collection and storage (e.g., Boswell et al. [5]; Unsworth et al. [48]). It will be important to clarify benefits for this group, such as how this approach could help support decision making with patients at risk of discontinuing or not benefiting from treatment or augmenting a clinician’s existing approach to formulation [5, 35]. It should also be made clear that algorithms used for clinical decision making do not replace the critical human relationship offered by a therapist [36]. We suspect that the best chance of clinician buy-in will happen when approaches to precision mental health are co-created with clinicians as well as people with lived experience. One final challenge is equipping clinicians with the knowledge and skills to use data and algorithm feedback. Ideally, this training would be available both in professional development settings and integrated into postgraduate qualifying programmes [5].

### Technical aspects

Another major hurdle to implementing routine outcome measurement for precision mental health in EDs is the need for robust and scalable software and hardware infrastructure. Clinical services will need to establish reliable networks for secure data storage and processing, which necessitates significant investment in on-site servers or cloud-based solutions to handle computational demands. Additionally, specialized hardware, such as electronic tablets or computers equipped with mental health software applications may be needed for collection and analysis of patient data. Incorporating these technologies into routine clinical workflows presents another challenge. The usability of systems, particularly the user interface and operational workflows, could be a barrier to adoption by clinicians and patients alike. Clinicians may resist integrating these technologies if the systems are perceived as inefficient or cumbersome to use. Moreover, the cost of technology, including both initial purchase and ongoing maintenance, can be prohibitive, particularly for smaller clinics or those lacking access to significant funding. This financial burden may further limit the widespread adoption of these technologies, making it challenging for clinics to implement precision medicine approaches without securing external funding or resources [18]. Obtaining research funding (as in the case of the U.K. EDCRN) is one approach but this is unlikely to offer a long-term sustainable solution.

### Statistics

While a detailed discussion of predictive algorithms is beyond the scope of this paper, a few aspects of Deisenhofer et al.’s [18] IPM deserve mention. First, model drift, which refers to the degradation of a model's predictive accuracy over time, causes a mismatch between the data the algorithm was based on and the data collected in clinic (Sahiner et al. [45]). This can happen due to changes in patient populations, treatment practices, or external factors like societal events. One illustrative example is the shift in quality and quantity of ED referrals during the COVID-19 pandemic [19]. Regular monitoring and recalibration of models would be required to mitigate drift as patient characteristics and treatment approaches evolve.

Additionally, generalizability of precision models is a significant statistical challenge, as many models are developed using data from specific settings, with reduced performance when applied to other settings [12]. Predictive models for EDs, like other mental health diagnoses, are likely initially to come from academic clinics or high-income countries, which limits their applicability in broader, more diverse clinical contexts. This lack of external validation and diverse representation means that models may fail to provide accurate predictions when applied to different populations or settings, such as primary care or underrepresented demographic groups. A related point is that predictive models require psychometrically sound measures that are suitable for the population they are being used with. The Eating Disorder Examination-Questionnaire (EDE-Q [22]) is one of the most widely used measures of ED psychopathology and is recommended for use in ICHOM [3], the Australian national minimum dataset [9], and the UK EDCRN [28]. However, the original 4-factor EDE-Q subscale structure has not been empirically supported (e.g., [1]) and it has taken several decades for appropriate norms to be identified for different groups (e.g., by age, sex-assigned-at-birth, gender identity, sexuality, ethnicity).

### Contextual factors

The implementation of precision treatment for EDs faces a range of contextual barriers that can complicate successful integration into routine clinical practice. One significant challenge is the applicability of predictive algorithms in rural and remote areas, under-represented and marginalised groups, and under-represented cultural regions. While large, research-oriented institutions likely have multi-disciplinary teams trained in a range of treatment approaches, clinicians in small, independent settings may be limited in the treatment modalities or levels of care available, limiting the applicability of precision suggestions. Similarly, data-driven decision-making may not generalise outside of the ‘WEIRD’ (Western, Educated, Industrialized, Rich, and Democratic) countries that are most commonly represented in research data. We may as a field need to think about the applicability of precision approaches in these settings, in comparison to approaches that fall more with the domain of personalised mental health.

Additionally, and as touched on above, precision treatment requires substantial upfront investment in technology development, data collection, and ongoing maintenance. Funding opportunities are limited everywhere but especially in lower-resource healthcare systems. Even when funding is available, the long-term financial sustainability of these systems remains uncertain, as they depend on continuous updates and integration into clinical practice. There are good practice examples of data collection and research being built into ‘business as usual’, even in non-academic settings (see positive practice examples, below), but not all areas will have the resources available to make this possible. Collaboration across regions and centres will be important for sharing what works (and what doesn’t) in different clinical contexts and in facilitating the pooling of resources and expertise where feasible.

Finally, the ethical considerations surrounding the use of data in precision medicine such as privacy concerns, potential for bias in algorithms, and the need for transparency in data handling, pose further barriers to widespread implementation. This is especially true given that some clinical outcome data, like the clinician-reported data captured in electronic health record systems, may be collected without explicit consent. Careful consideration is required around the ethics of ‘broad consent’ for phenotyping from biological data (including biomarker, genetic and other biological data) and secondary use of clinical data. We should also be particularly mindful of potential bias in algorithms given the existing documented biases within ED research, such as the under-reporting of race and ethnicity and mainly White participants within clinical trials [21, 24], the impact of race and ethnicity on ED recognition (e.g., Clayton Erickson et al. [13]); the lack of understanding and data on ED symptom presentations more common in males [38] and sexual and gender minority groups (e.g., [40]), and the importance of intersectionality in understanding ED experiences and symptom profiles (e.g., [7]). As argued by Deisenhofer et al. [18] the collection of more diverse data would support the minimization of these biases.

### Positive practice examples

There are examples of well-implemented routine data collection which have potential to inform precision treatment. In the UK, the Increasing Access to Psychological Therapies (IAPT) programme (now known as Talking Therapies) embedded systematic use of routine outcome monitoring with digital systems to support this. Session-by-session measures include the Patient Health Questionnaire-9 measure of depression (PHQ-9), Generalized Anxiety Disorder Scale-7 for anxiety (GAD-7) and the Work and Social Adjustment Scale (WSAS), with other disorder-specific questionnaires used as appropriate [49]. This routine outcome monitoring system has led to England-wide evaluation of Talking Therapies services, yielding insight into factors that lead to positive (and less positive) treatment outcomes and more (and less) effective services. A meta-analysis of 60 studies was able to draw on evaluations from Talking Therapies services with sample sizes ranging from n = 1 (single-case study) up to n = 537,131 [49]. Notably, the Talking Therapies approach has led to similar initiatives being introduced in Australia (e.g., Cromarty et al. [15]), Canada (Naeem et al. [39]), Japan (Kobori et al. [31]) and Norway (Knapstad et al. [30]), highlighting that learning can extend across geographical regions and different healthcare systems.

Thinking about ED services specifically, the Western Australian Centre for Clinical Interventions (CCI) has successfully embedded routine outcome measurement to allow for outcome evaluation in the majority of patients they see. The CCI service offers treatment for depression and anxiety as well as EDs and has published over 200 peer-reviewed articles over 20 years. In 2022, the service produced a research strategy based on the core premise that research and evaluation are fundamental components of the CCI model of care [11]. Their research is supported through collaboration between researchers, clinicians and people with lived experience and aims to optimise clinical outcomes within CCI while also having a positive impact within Australia and internationally. In 2011, the CCI ED service published the first routine evaluation of Enhanced CBT for EDs (CBT-E) [10], drawing on data from 125 patients. This was a comparable sample size to the seminal randomised controlled trial of CBT-E (N = 149 participants) [23]. Most CCI studies have used traditional pre/post-treatment designs, but with learning from precision medicine, these evaluations may be expanded to allow successful application of session-by-session data to personalised treatment decisions.

## Future directions

A key aspect of precision mental health that the ED field has yet to successfully traverse is the integration of biological data. Biomarkers used in other areas of precision mental health include data from genetic, microbiome, inflammatory, and neuroimaging sources. As a group, we seem particularly enthusiastic to capitalize on use of genomic testing in research, with its recent technological advances and lowered costs (see [6] for an overview of the global Eating Disorder Genetics Initiative). However, the ED field has not yet implemented biomarker data collection in routine clinical services [41]. Alongside moving forward with the biological components of precision treatment, we should be mindful of the growing awareness of social determinants of health in EDs [34]. Social determinants of health are the non-medical factors that influence health outcomes, such as housing, food insecurity, and exposure to discrimination. Glasgow et al. [26] highlight that linking social determinants of health to other forms of data, including biomarkers, may offer the most potential when using a precision approach. This is consistent with the much-cited biopsychosocial model, which is generally understood as key in EDs, but is often considered with a primary emphasis on psychological or psychosocial factors and with research embedded in ‘WEIRD’ (Western, Educated, Industrialized, Rich, and Democratic) contexts.

## Clinical implications

The above suggested ideas present an ambitious path forward for precision mental health in EDs, with potential for long-term and far-reaching clinical implications. In the immediate term, however, they may seem far removed from daily clinical practice. As such, we have included initial ideas that clinicians interested in personalised and precision mental health via routine outcome measurement may choose to implement (see Table 1).

**Table 1:** Small movements toward personalised and precision mental health in EDs via routine outcome measurement

• Add an interim timepoint to your pre/post measurements to reduce “lost” cases (e.g., week 4–6 of treatment) • If you are already collecting patient data, share that data with your patient • Increase the duration of follow-up measurement • Track other DSM/ICD categories beyond EDs to identify transition to other mental health diagnoses, where feasible, e.g., via electronic health records or a checklist approach • Incorporate the social determinants of health most relevant to your setting into your measurement approach • Explore whether your current measures have evidence for sensitivity to change. If not, consider if you and your team may be well placed to create this evidence for the ED field	

## Conclusion

The promises of precision mental health bring new hope to the ED field. There are now three available frameworks for routine data collection in clinical ED services (ICHOM, Australian national minimum dataset, U.K. EDCRN), all of which have emerged in the last 2 years. These frameworks have all been developed using co-production methodologies with people with lived experience, clinicians and researchers but, given their recency, there is little to no published work on the piloting or implementation of these approaches. Everyone working in the field of EDs can benefit from taking steps towards personalised and precision mental health but barriers to implementation will require consideration across stakeholders and settings. Continued collaboration between people with lived experience and families, clinicians and researchers is key. Consideration of evidence from diverse settings and contexts is also needed, including individual case examples and single service explorations on the feasibility and acceptability of comprehensive routine outcome measurement, through to large-scale collaborations facilitating complex data analysis and algorithmic approaches. Large, well-funded centres may need to serve as exemplar sites that can then offer learning and implementation support to smaller, less well-resourced settings. Ultimately, precision medicine algorithms will be most accurate and helpful when drawing on data from a wide range of patients and settings, which highlights the importance of collaboration between centres and may support sharing of resources where appropriate.

## Data Availability

Not applicable
